# Extracellular Vesicles Released by Oxidatively Injured or Intact C2C12 Myotubes Promote Distinct Responses Converging toward Myogenesis

**DOI:** 10.3390/ijms18112488

**Published:** 2017-11-22

**Authors:** Michele Guescini, Serena Maggio, Paola Ceccaroli, Michela Battistelli, Giosuè Annibalini, Giovanni Piccoli, Piero Sestili, Vilberto Stocchi

**Affiliations:** Department of Biomolecular Sciences, University of Urbino Carlo Bo, Via I Maggetti, 26, 61029 Urbino, Italy; serena.maggio@uniurb.it (S.M.); paola.ceccaroli@uniurb.it (P.C.); michela.battistelli@uniurb.it (M.B.); giosue.annibalini@uniurb.it (G.A.); giovanni.piccoli@uniurb.it (G.P.); piero.sestili@uniurb.it (P.S.); vilberto.stocchi@uniurb.it (V.S.)

**Keywords:** exosomes, extracellular vesicles, oxidative stress, myogenic differentiation, intercellular communication, C2C12, H_2_O_2_

## Abstract

Myogenic differentiation is triggered, among other situations, in response to muscle damage for regenerative purposes. It has been shown that during myogenic differentiation, myotubes release extracellular vesicles (EVs) which participate in the signalling pattern of the microenvironment. Here we investigated whether EVs released by myotubes exposed or not to mild oxidative stress modulate the behaviour of targeted differentiating myoblasts and macrophages to promote myogenesis. We found that EVs released by oxidatively challenged myotubes (H_2_O_2_-EVs) are characterized by an increased loading of nucleic acids, mainly DNA. In addition, incubation of myoblasts with H_2_O_2_-EVs resulted in a significant decrease of myotube diameter, myogenin mRNA levels and myosin heavy chain expression along with an upregulation of proliferating cell nuclear antigen: these effects collectively lead to an increase of recipient myoblast proliferation. Notably, the EVs from untreated myotubes induced an opposite trend in myoblasts, that is, a slight pro-differentiation effect. Finally, H_2_O_2_-EVs were capable of eliciting an increased interleukin 6 mRNA expression in RAW264.7 macrophages. Notably, this is the first demonstration that myotubes communicate with surrounding macrophages via EV release. Collectively, the data reported herein suggest that myotubes, depending on their conditions, release EVs carrying differential signals which could contribute to finely and coherently orchestrate the muscle regeneration process.

## 1. Introduction

Skeletal muscle is a highly plastic tissue capable of adapting to different stresses, in part due to its remarkable regenerative capacity. This feature is largely attributable to the presence of satellite cells, which are undifferentiated mononucleated muscle precursors located beneath the basal lamina of myofibers [1,2]. In response to different stimuli (injury, stretch, exercise, etc.), satellite cells exit from G_0_-quiescent state, re-enter the cell cycle and proliferate. After several rounds of proliferation, the majority of cells differentiate and fuse to form new myofibers or to repair damaged ones, while the remaining cells return to quiescence replenishing the initial satellite cell population [3].

Regeneration represents a highly coordinated process in which satellite cells are activated to maintain and preserve tissue structure and function. Many factors, such as local or systemic growth factors and inflammatory mediators, have been shown to be involved in the regulation of myoblasts’ proliferation and differentiation to promote muscle repair or regeneration [4].

In addition to soluble proteins, it has recently been found that C2C12 myoblasts and myotubes also release exosome-like vesicles in the extracellular environment [5,6,7]. Extracellular vesicles (EVs) are spherical structures bound by a membrane similar in composition to that of the cell from which the vesicle was originated. Their contents include a variety of cytoplasmic elements which are also indicative of their cell of origin [8]. EVs can be taken up by other cells, thus modulating the activity of recipient cells in vitro [9,10,11] and in vivo [12,13,14]. Biochemical techniques have been used to identify protein, mRNA, miRNA, DNA and lipid contents of EVs [15,16,17,18,19,20]. Among these, for example, EVs contain a collection of membrane proteins such as G protein-coupled receptors, integrins, major histocompatibility complex I and II, transferrin receptors and tetraspanins [19,21], which can activate downstream signalling pathways in target cells, such as calcium signalling [22], mitogen-activated protein kinase activation [23] or Fas signalling [24]. 

Moreover, recent evidence demonstrated that EVs may transfer to target cells genetic information, in particular, microRNA (miRNA) [15,16,20,25,26,27] and DNA [17,28,29]. miRNA are a class of 20–22 nucleotide non-coding RNAs that regulate gene expression in mammals at the post-transcriptional level [30]. Notably, recent studies have shown that muscle-specific miRNAs mediate the regulation of muscle cell proliferation, differentiation, contractility and stress responsiveness [31], for example, miR-133a, -206 and -1 affect muscle cells, regulating gene expression and signalling mediators involved in muscle development and function [32,33,34,35]. Our and other reports opened up the possibility that EVs convey signalling molecules allowing for intercellular regulation of gene expression [5,25,36]. This early hypothesis has recently been confirmed and extended by findings showing that exosome-like vesicles released by myotubes may have a role in promoting the downregulation of cyclin D1 and sirt1, and the upregulation of myogenin in myoblasts [37,38]. Despite these findings on the secretory function of muscle cells, the biological roles of skeletal muscle EVs are not well understood yet, and more importantly, there are no studies on the role of EV-mediated communication in response to oxidative injuries.

The aim of this study was to investigate whether the release of extracellular vesicles from oxidatively challenged myotubes in the microenvironment could modify the behaviour of surrounding myoblasts and macrophages, two of the key players in the muscle niche.

Herein we present new evidence that EVs from control or oxidatively stressed myotubes exert differential effects on the recipient, differentiating myoblasts and showing a tendency to modulate an inflammatory response in RAW264.7 macrophages. These new findings will help to shed light on the mechanisms underlining intercellular communication during muscle regeneration and repair.

## 2. Results

### 2.1. Oxidative Treatment of C2C12 Myotubes

Previous studies by our and other groups have shown that a mildly toxic oxidative stress is capable of negatively affecting the execution of the myogenic program without causing significant cell demise. Such a situation may be representative of a physio/pathological situation occurring in the course of moderate inflammation, such as that arising following intense physical exercise or muscular overstretches resulting in fiber damage.

**Figure 1:**
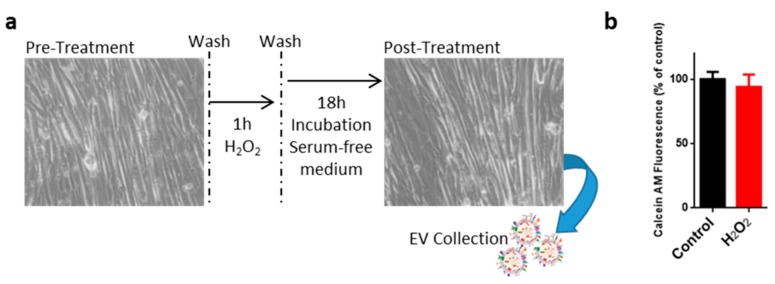
Exposure of myotubes to oxidative stress. C2C12 myotubes were treated with 0.3 mM H_2_O_2_ for 1 h and then extracellular vesicles (EVs) were collected after 18 h of recovery (blue arrow) (**a**); cell viability was evaluated after the collection of EVs using calcein AM, the results are represented as mean ± standard deviation (SD); *n* = 3 (**b**).

In this study, the C2C12 cell line was used as a model to investigate the possible role of EVs in mediating communication between injured myotubes and surrounding myoblasts in response to exposure to an oxidative stressor such as H_2_O_2_. To this end, myotubes at the fifth day of differentiation have been exposed to a brief (1 h) challenge with 0.3 mM H_2_O_2_; after 18 h of recovery, conditioned medium was collected and EVs were purified using a serial ultracentrifugation procedure (Figure 1a). In order to exclude the release of cellular debris due to extensive cell death, cell viability was evaluated 24 h after oxidative injury. As shown in Figure 1b, the oxidative challenge did not result in a significant reduction of viable myotubes. These data confirm previous evidence showing that mild oxidative stress does not affect C2C12 myotubes’ viability [39].

### 2.2. Characterization of EVs Released after H_2_O_2_ Oxidative Stress

**Figure 2:**
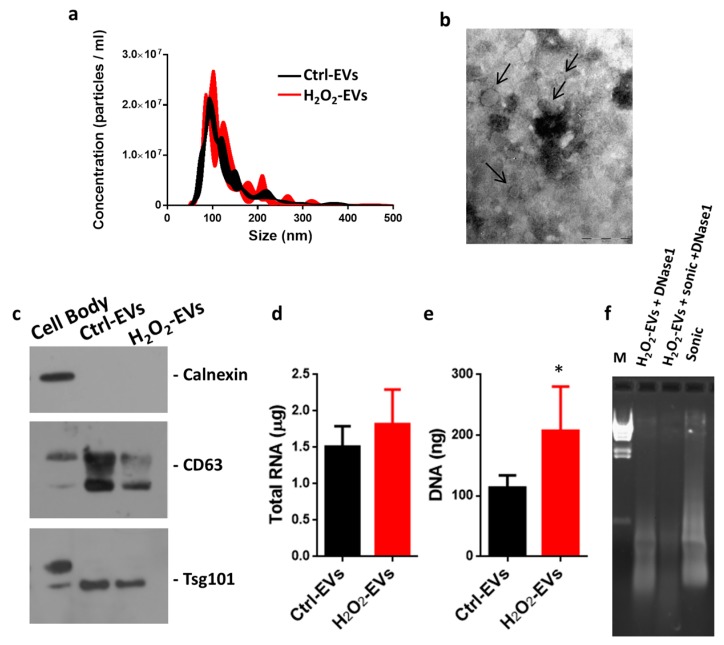
Characterization of EVs released by myotubes following oxidative challenge. EVs released by oxidatively injured (H_2_O_2_-EVs) and intact (Ctrl-EVs) C2C12 myotubes were analysed and counted using a nanoparticle tracking assay, and results are represented as mean ± SEM; *n* = 4 (**a**); transmission electron microscopy analysis of EVs released by oxidatively injured myotubes, the arrows indicate the presence of small vesicles of about 30–100 nm in diameter (bar 200 nm) (**b**); Western blot analysis of myotube cell bodies, Ctrl-EVs and H_2_O_2_-EVs; blots were probed with antibodies against calnexin, CD63 and Tsg101 (**c**); total RNA (**d**) and DNA (**e**) contained in EVs were quantified using RiboGreen™ and PicoGreen™ (molecular probes), respectively; results are represented as mean ± SD; *n* = 3; * *p* < 0.05. The DNA carried by H_2_O_2_-EVs was analyzed using electrophoresis migration after DNase1 digestion with and without disruption of the membranous envelope by sonication (**f**).

The nanoparticle tracking assay of EVs purified following H_2_O_2_ treatment showed that the distribution plot of particle hydrodynamic diameter was not significantly different in control versus treated conditions, with a mode of size distribution of 99 ± 11 nm and 90 ± 13 nm, respectively. Furthermore, the exposure of myotubes to 0.3 mM H_2_O_2_ failed to elicit an increased release of EVs (Figure 2a). Transmission electron microscopy (TEM) showed that EVs released by H_2_O_2_-treated myotubes (H_2_O_2_-EVs) appeared as rounding vesicles delimited by an intact, dense outer wall and a less dense inner region, with a mean diameter of approximately 57 nm (Figure 2b); these data are in agreement with the exosomes’ features reported in the literature [40]. 

Since H_2_O_2_ treatment did not induce apparent morphological modifications of released EVs, biochemical characterization of particles was then undertaken. Western blot analysis showed that control and H_2_O_2_-EVs were positive for CD63 and Tsg101, two well-defined exosome markers, and negative for calnexin, a marker of the endoplasmic reticulum (Figure 2c). On the whole, these data confirmed that in our conditions, regardless of the imposition of oxidative stress, the isolated vesicles were mainly exosomes. Nucleic acid quantification, interestingly, showed that there was a slight but not significant increase in total RNA amount in H_2_O_2_ conditions versus control (Figure 2d), whereas a higher level of DNA was found in H_2_O_2_-EVs (Figure 2e). These data suggest that mild oxidative stress induces cellular events that result in increased EV loading of nucleic acids (mainly DNA) but do not seem to alter the main features of the vesicles. To further investigate if DNA was contained within vesicles, we performed electrophoretic analysis using nucleic acids purified from H_2_O_2_-EVs after DNase1 treatment. Figure 2f shows that the DNase1 treatment of intact EVs was unable to completely digest DNA, whereas adding a sonication step caused the DNA fragments to disappear. This highlighted that these DNA fragments were entirely wrapped by membranous structures.

### 2.3. H_2_O_2_-EVs Promote Proliferation of Differentiating Myoblasts

**Figure 3:**
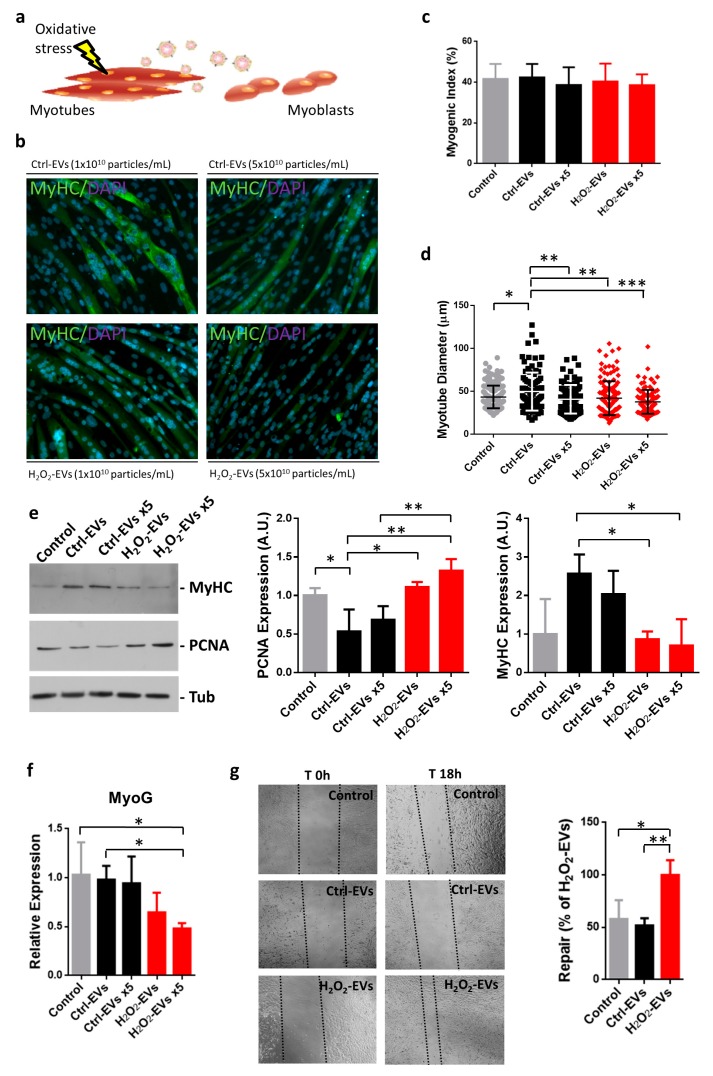
Treatments of myoblasts with EVs released by myotubes following oxidative challenge. Schematic representation of myoblast treatments with EVs (**a**); myoblasts were treated using 1 × 10^10^ particles/mL (indicated as Ctrl-EVs or H_2_O_2_-EVs) or using 5 × 10^10^ particles/mL (indicated as Ctrl-EVs × 5 or H_2_O_2_-EVs × 5). Immunofluorescence analysis was performed 4 days after EV treatments (**b**); the obtained differentiation index (**c**), and the myotube size (**d**), were reported (*n* = 3, see Materials and Methods section). Myosin heavy chain (MyHC), proliferating cell nuclear antigen (PCNA) and tubulin (Tub) protein expression (**e**) and myogenin (MyoG) mRNA expression (**f**) analyses were carried out 24 h after EV addition; the results are represented as mean ± SD; *n* = 3; * *p* < 0.05; ** *p* < 0.01; *** *p* < 0.001. Migration of C2C12 cells after wounding: confluent cultures of C2C12 myoblasts were wounded with a sterile blade (*T* = 0 h), deprived of serum and treated with Ctrl-EVs or H_2_O_2_-EVs for 18 h (*T* = 18 h), then recolonization was quantified by light microscopy (magnification 40×; Zeiss) and reported as percentage of the higher one; the results are represented as mean ± SD (*n* = 4); * *p* < 0.05; ** *p* < 0.01 (**g**).

We hypothesized that injured myotubes could modulate their own microenvironment sending “help” signals in the form of EVs to surrounding myocytes. In this view, to determine whether H_2_O_2_-EVs could be involved in the regulation of C2C12 myogenic differentiation, EVs harvested from the cultured media of control or oxidatively stressed myotubes were added (EV concentrations were comparable to, or five times higher than, that of the medium at the end of the harvest stage) to myoblasts committed to differentiate by serum withdrawal (Figure 3a). 

The fusion index analyzed in differentiating control myocytes and in myocytes treated with Ctrl-EVs or H_2_O_2_-EVs was of about 40% in all tested conditions, demonstrating that both of these EVs were not involved in the regulation of fusion processes during the early phase of myogenic differentiation (Figure 3b,c).

More interestingly, our data showed that myotube diameter was significantly affected by EV treatments. Ctrl-EVs induce a slight but significant increase in myotube diameter; on the contrary, H_2_O_2_-EVs lack this effect, but promote a slight decrease of myotube size. Consequently, the diameter size of Ctrl-EV and H_2_O_2_-EV myotubes was significantly different, and this difference increased at higher H_2_O_2_-EV concentrations (Figure 3d). Moreover, Figure 3d shows that increasing Ctrl-EV levels failed to induce hypertrophic stimulation compared to lower levels of Ctrl-EVs, a finding in agreement with recent evidence showing that high EV levels may impair differentiation in adipose-derived stem cells [41]. The modulation of the myogenic differentiation process in response to H_2_O_2_-EV treatment was further investigated by Western blot and gene expression analyses. Protein expression data showed an upregulation of myosin heavy chain (MyHC), an affordable marker of myogenic differentiation, in Ctrl-EVs compared with H_2_O_2_-EV conditions (Figure 3e); at the same time, PCNA protein expression, a proliferation marker, was downregulated in response to Ctrl-EV and upregulated following H_2_O_2_-EV treatments (Figure 3e). Moreover, MyoG mRNA expression levels revealed a significant downregulation in H_2_O_2_-EV treatments (Figure 3f). To further support the supposed ability of H_2_O_2_-EVs to elicit myoblast proliferation, a wound assay was set up; as shown in Figure 3g, H_2_O_2_-EV treatment induced myoblast proliferation that resulted in an almost complete repair of the wound.

Altogether, these data suggest that Ctrl-EVs could be involved in the regulation of cell cycle exit and stimulation of myogenic differentiation. More interestingly, H_2_O_2_-EVs seem to carry a different “message”, which could trigger a shift of the balance of proliferation/differentiation towards proliferation.

### 2.4. H_2_O_2_-EVs Induce Interleukin 6 Expression in RAW264.7 Macrophages

**Figure 4:**
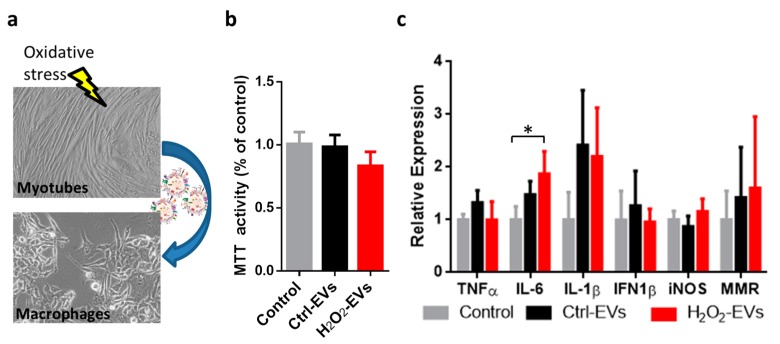
Treatments of RAW264.7 macrophages with EVs released by myotubes following oxidative challenge. The blue arrow indicates the addition of myotube-derived EVs to RAW264.7 cells. RAW264.7 macrophages were treated using 1 × 10^10^ particles/mL from control (Ctrl-EVs) or oxidatively injured (H_2_O_2_-EVs) myotubes (**a**); MTT cell viability assay (**b**) and mRNA expression (**c**) analyses were performed 24 h after EV treatments. The results are represented as mean ± SD; *n* = 4; * *p* < 0.05.

Recent discoveries have shown that the immune system response to muscle injury is a complex regulated process involving multiple myeloid cell populations which regulate one another’s function, as well as the regeneration of muscle. In order to evaluate whether H_2_O_2_-EVs released by oxidatively injured myotubes could also mediate muscle–macrophage communication, myotube-derived EVs were used to treat RAW264.7 cells, a suitable cell line model of macrophages (Figure 4a). The concentration of EVs used in these experiments was non-toxic to RAW264.7, as shown by the results of an MTT assay performed 24 h post-treatment (Figure 4b). The mRNA expression analysis of key macrophage markers showed that after treatment, IL-6 was upregulated in response to H_2_O_2_-EVs, while a less-marked stimulation was obtained using Ctrl-EVs; IL-1β showed only a trend to increase in both conditions, whereas all the other markers were unaffected by EV treatments (Figure 4c).

## 3. Discussion

Skeletal muscle has the capacity to regenerate following common use-related injuries. Such events are accompanied, as a rule, by inflammatory processes whose persistence may imbalance the repair task. Oxidative stress, the occurrence of which is universally recognized during inflammation, has been shown to affect the proliferation/differentiation balance of satellite-derived cells and myoblasts [42,43,44] and to promote the loss of mature myofibers [45]. Furthermore, it is known that oxidative stress results in proliferation/differentiation imbalance playing a concausal role in many myopathies such as Duchenne dystrophy [46], myotonic dystrophy [47], sarcopenia [48] and cachexia [45].

Although the deleterious effects of reactive oxygen species on muscle homeostasis are well documented, to the best of our knowledge there are very few data in the literature specifically dealing with the modulation of the muscle stem cell niche by means of EVs under oxidative stress.

EVs are exciting candidates for novel signalling factors in stem cell niches [49,50,51]; in fact, EVs are able to carry complex signals to target cells, thus contributing to modifying the microenvironment. Pioneering studies have demonstrated that EVs released from embryonic stem cells are capable of altering the expression of genes in neighbouring cells [25]: for example, Ratajczak et al. [52] demonstrated that the EVs released by embryonic stem cells stimulate the proliferation of hematopoietic progenitors.

Skeletal muscle cells secrete a large number of myokines and EVs that influence the growth, function and development of muscle tissue [5,6,53]. Increasing effort is being devoted to ascertain the precise role of EVs in controlling cell fate and promoting skeletal muscle regeneration.

Cellular state and changes in the cell microenvironment can widely affect EV composition; in fact, stress situations such as starvation, hypoxia or oxidative stress can alter EV content, and thus the messages they carry. Interestingly, several reports show that exosomes released under stress situations can confer protection on recipient cells [54]. For example, oligodendrocytes secrete exosomes that are taken up by neurons and improve neuronal survival under conditions of oxidative stress or starvation [55]. Additionally, cardiac progenitor cells release exosomes that promote tissue vascularization and angiogenesis, and display cardio-protective effects during ischemia/reperfusion injury through reduction of oxidative stress and activation of prosurvival signals [56]. Along this line, further comments can be drawn: H_2_O_2_ acts hormetically in a number of biologically relevant processes, including myoblast regeneration and wound repair. In general, hormesis means that low doses of a given agent stimulate physiological responses, while higher doses trigger toxic ones: in the case of H_2_O_2_, the situation is even more complicated since, for instance, the same dose acting for different time lengths in different tissues may elicit diverging responses [57]. Thus, a fine-tuning of these subtle but substantial differences—maybe more complex than is generally accepted—should exist, and indeed we show that EVs secreted under mildly oxidatively toxic conditions (H_2_O_2_-EVs) subsequently serve as carriers of pro-proliferative responses. 

Our data obtained analysing the effect of EVs released by normal or stressed myotubes confirm and further extend this view. 

Indeed, the results illustrated herein suggest that EVs released by oxidatively challenged myotubes stimulate serum-withdrawn myoblasts’ proliferation. Since we and others previously demonstrated that oxidative stress inhibits myoblast proliferation and impairs the myogenic process [43], it is of worth that the present data suggest a protective role of H_2_O_2_-EVs; indeed, H_2_O_2_-EVs shifted recipient myoblasts toward proliferation and potentially prevented the proliferation/differentiation imbalance caused by the oxidant. 

In parallel, equally in line with current evidence, we also show that Ctrl-EVs favour myogenic differentiation as assayed with multiple and integrated approaches.

These two sets of data suggest that, depending on the conditions of the releasing myotubes, EVs may carry specific and distinct signals to surrounding cells, thus contributing to the fine tuning of “microenvironment instructions”. 

This microenvironment contains several cell types, including not only myotubes and myogenic cells but also endothelial, mesenchymal-like, and myeloid cells such as hematopoietic-like cells, neutrophils and macrophages [58,59]. In particular, with regard to the latter of these, recent investigations demonstrate that myeloid cells play a central role in muscle regeneration. Interestingly, the present findings show that H_2_O_2_-EVs can stimulate RAW264.7 macrophages to express higher levels of IL-6. 

It is of worth that IL-6 released by neutrophils and M1 macrophages has a strong influence on the normal progression of the proliferative stage, and seems to be necessary for the transition into the early differentiation steps [3,60] contributing to the commencement of the regenerative phase.

Although our data do not provide a mechanistic breakthrough, a tempting scenario of the condition-dependent effects described herein might point to myogenin downregulation—which favours the maintenance of a proliferative phenotype—and IL-6 overexpression—which promotes myogenic and repair responses.

The interaction with specific signalling cascades might also be involved in the above responses: for example, the activation of TGFβ or BMP signalling might downregulate myogenin expression, while IL-6 release might lead to JAK/STAT signalling pathway activation promoting myoblast proliferation and/or differentiation [61]. Moreover, EVs released by injured myotubes may act as chemotactic signals, recruiting macrophages which eliminate from the microenvironment potentially harmful cellular debris and byproducts; at the same time, macrophages play a proactive role in myogenesis, releasing specific chemokines, a phase which is then followed by a shift toward an anti-inflammatory, non-phagocytic and pro-differentiation phenotype [62].

Given the pathophysiological implications of these events, further studies aimed at elucidating the mechanisms responsible for these effects will be needed and deserve consideration.

## 4. Materials and Methods

### 4.1. Cell Cultures, H_2_O_2_ Treatement, Viability and Wound Healing Assays

RAW264.7 cells and C2C12 mouse adherent myoblasts were cultured in Dulbecco’s Modified Eagle Medium(DMEM) supplemented with 10% heat-inactivated fetal bovine serum (FBS), 2 mM glutamine, penicillin (100 U/mL) and streptomycin (100 μg/mL), and maintained in a 5% CO_2_ atmosphere at 37 °C. To induce myogenic differentiation of C2C12 myoblasts, when about 80–90% cell confluence was attained, the 10% fetal bovine serum was changed with 2% horse serum, as previously described [63]. After 5 days of differentiation, C2C12 myotubes were oxidatively challenged with 0.3 mM H_2_O_2_ for 1 h in fresh serum-free RPMI 1640 medium. Cells were then extensively washed with phosphate buffered saline (PBS) (8 g/L NaCl, 1.15 g/L Na_2_HPO_4_, 0.2 g/L KH_2_PO_4_, 0.2 g/L KCl) and incubated in serum-free DMEM for 18 h. To assess cell viability, the membrane-permeable dye calcein AM was prepared as a stock solution of 10 mM in dimethylsulfoxide and used at a concentration of 0.1 µM in serum-free DMEM. Cells were incubated with calcein AM working solution at 37 °C in the dark for 30 min, and washed in PBS for immediate analysis of calcein fluorescence retention in cells using Fluoroskan Ascent FL (Thermo, Waltham, MA, USA). Results are expressed as percent fluorescence versus control samples. For the wound healing assay, confluent C2C12 cells, wounded with a sterile blade, were incubated for 18 h at 37 °C, 5% CO_2_, and the recolonization was quantified by light microscopy (Zeiss, Oberkochen, Germany).

### 4.2. Extracellular Vesicle Isolation

Conditioned medium from 5 × 10^7^ cells was collected 18 h after H_2_O_2_ treatment. Extracellular vesicles were purified by centrifugation for 15 min at 1000× *g* and then for 15 min at 2000× *g* to eliminate cell debris. Supernatants were further centrifuged for 20 min at 18,000× *g*. The resulting supernatants were pelleted by ultracentrifugation at 110,000× *g* for 70 min. The EV pellets were washed in 3 mL PBS, centrifuged again and resuspended in PBS.

### 4.3. Nanoparticle Tracking Analysis

EVs were diluted to approximately 1 mL of PBS, loaded into the sample chamber of an LM10 unit (Nanosight, Malvern, UK) and three videos of either 30 or 60 s were recorded of each sample. Analysis was performed with NTA 3.1 software (Nanosight) and data are presented as the mean ± SD of the three video recordings. When samples contained high numbers of particles, they were diluted before analysis and the relative concentration was then calculated according to the dilution factor. Control 100 and 400 nm beads were supplied by Malvern Instruments Ltd. (Malvern, UK). 

### 4.4. Transmission Electron Microscopy

For a detailed morphological analysis, specimens were processed with transmission electron microscopy observation using the conventional negative staining procedure. EVs isolated from C2C12 culture media were adsorbed to formvar carbon-coated 200 mesh grids (Agar Scientific Ltd., Stansted, UK) for 2 min, and gently washed with filtered PBS. EVs on grids were immediately fixed with 2.5% glutaraldehyde for 1 min. The grids were incubated with 2% (*w*/*v*) sodium phosphotungstate for 1 min and the liquid excess was removed with filter paper. After negative staining, specimens were observed by means of a Philips CM10 transmission electron microscope at 80 kV.

### 4.5. Nucleic Acid Quantification and DNase1 Digestion

The DNA and total RNA contained within EVs were quantified using Quant-iT™ PicoGreen™ dsDNA Assay Kit and Quant-iT™ RiboGreen™ RNA Assay Kit, respectively, according to the manufacturer’s instructions. The presence of DNA enwrapped within EVs was confirmed by DNase1 digestion (Promega, Madison, WI, USA) for 15 min at 37 °C with or without 3 rounds of 10 s sonication.

### 4.6. Western Blotting Analysis

Protein extracts were obtained from the organic phase after Qiazol cell lysis following Qiagen User Protocol RY16 May-04. The protein pellet was dissolved in ISOT buffer (8 M urea, 4% CHAPS (3-[(3-Cholamidopropyl)dimethylammonio]-1-propanesulfonate hydrate), 65 mM DTE (1,4-Dithioerythritol), 40 mM Tris base and added with SIGMAFAST™ Protease Inhibitor Cocktail (Sigma-Aldrich, Milan, Italy)) and sonicated for 5 s on ice. For electrophoresis, samples containing 10–30 µg were mixed with the Laemmli sample buffer 4× (1:4 ratio) and loaded onto 10% SDS–PAGE gels. The proteins were then blotted to a PVDF (Polyvinylidene difluoride) membrane (Thermo). Primary antibodies were used against CD63 (1:500 dilution, clone sc-5275 (MX-49.129.5) Santa Cruz, CA, USA), Tsg101 (1:1000 dilution, clone Sigma-Aldrich T5701) and calnexin (1:2000 dilution, clone Sigma-Aldrich C4731). Primary antibodies were incubated overnight at 4 °C followed by washing and the application of secondary HRP-conjugated antibody (Pierce, Waltham, MA, USA), and the immune complexes were visualized using the Clarity and/or Clarity Max (Bio-Rad, Milan, Italy), and the obtained auto-radiographic films were quantified using ImageJ software.

### 4.7. Immunofluorescence Assays and Myotube Analysis

Cells were fixed for 15 min at room temperature using 4% formaldehyde/PBS and permeabilized in 0.5% Triton X-100/PBS for 4 min. Expression of MyHC was assayed using the mouse monoclonal antibody ascite against myosin (MF20, obtained from DSHB) at the 1:2 dilution and incubated for 1 h at 37 °C followed by a 30-min incubation at 37 °C with 1:100 fluorescein-conjugated goat anti-mouse IgG (Biolegend, San Diego, CA, USA). Cells were stained with DAPI (2-(4-amidinophenyl)-1*H*-indole-6-carboxamidine) for nuclear visualization and mounted in Mowiol 4-88 (Sigma-Aldrich). Images were acquired with IM50 software (Leica, Wetzlar, Germany) using a DC300F digital camera connected to a Leica microscope. Fusion index was determined by counting the number of nuclei in differentiated myotubes and expressed as a percentage of the total number of nuclei. For each experimental condition, 1000 nuclei were counted in three independent cultures. Myotube size was estimated measuring the diameter of at least 100 myotubes using ImageJ software. The average diameter per myotube was calculated as the mean of five measurements taken along the length of the myotube.

### 4.8. Gene Expression Analysis

Total RNA was extracted from differentiating C2C12 myoblasts and RAW264.7 macrophages. The RNA purification was performed using the E.Z.N.A.^®^ Total RNA Kit I (Omega Bio-tek, Norcross, GA, USA) according to the manufacturer’s instructions; to eliminate contaminant DNA, DNase I digestion (Ambion, Paisley, UK) was performed. cDNA was obtained using the Maxima Reverse Transcriptase kit (Thermo). Real-time PCR amplifications were conducted using Sensi-FAST SYBR Green (Bioline USA Inc., Taunton, MA, USA), with 300 nM primers. Specific primers used: myogenin (MyoG-F: 5′-GCA CTG GAG TTC GGT CCC AA-3′ and MyoG-R: 5′-TTG TGG GCG TCT GTA GGG TC-3′); TNFα (TNFα-F: 5′-GCT CTT CTG TCT ACT GAA CTT CGG-3′ and TNFα-R: 5′-ATG ATC TGA GTG TGA GGG TCT GG-3′); IL-6 (IL-6-F: 5′-AGC CAG AGT CCT TCA GAG AGA TAC-3′ and IL-6-R: 5′-AAT TGG ATG GTC TTG GTC CTT AGC-3′); IL-1β (IL-1β-F: 5′-TGA CGT TCC CAT TAG ACA ACT G-3′ and IL-1β-R: 5′-CCG TCT TTC ATT ACA CAG GAC A-3′); interferon beta 1 (IFN1β) (IFN1β-F: 5′-TCC AAG AAA GGA CGA ACA TTC-3′ and IFN1β-R: 5′-TGA GGA CAT CTC CCA CGT CAA-3′); inducible nitric oxide synthase (iNOS) (iNOS-F: 5′-CCC TTC CGA AGT TTC TGG CAG CAG C-3′ and iNOS-R: 5′-CCA AAG CCA CGA GGC TCT GAC AGC C-3′); mannose membrane receptor (MMR) (MMR-F: 5′-CAT GAG GCT TCT CCT GCT TCT G-3′ and MMR-R: 5′-TTG CCG TCT GAA CTG AGA TGG-3′); S16 (S16-F: 5′-TGA AGG GTG GTG GAC ATG TG-3′ and S16-R: 5′-AAT AAG CTA CCA GGG CCT TTG A-3′); and GAPDH (GAPDH-F: 5′-TCA ACG GCA CAG TCA AGG-3′ and GAPDH-R: 5′-ACT CCA CGA CAT ACT CAG C-3′). Thermocycling was conducted using a LightCycler 480 (Roche, Basel, Switzerland) and started by a 2 min incubation at 95 °C, followed by 40 cycles (95 °C for 5 s; 60 °C for 5 s; 72 °C for 10 s). Each reaction was conducted in triplicate, and melt-curve analysis was used to confirm the specificity of each amplification and lack of primer dimers. Relative quantification of mRNA expression levels was performed according to the Δ*C*_q_ method, and the expression levels of GAPDH and S16 were used as a reference [64].

### 4.9. Statistical Analysis

Statistical analyses were performed using GraphPad Prim version 6 statistical software (GraphPad Software, San Diego, CA, USA). Unless noted otherwise, the results are expressed as the mean ± SD. The statistically significant differences between the mean values recorded for each experimental condition was calculated by Student’s *t*-test or ANOVA analysis (Tukey’s multiple comparison test was used as post-hoc) and *p*-values are indicated where appropriate in the figures and their legends. Statistical significance was indicated as follows: * *p* < 0.05; ** *p* < 0.01; *** *p* < 0.001.

## 5. Conclusions

Within the muscular niche, after a damaging/stressing situation, different cells characterized by different lineages or by specific conditions, that is, normal or stressed, regenerate. Among these cells, normal or oxidatively injured myotubes play a pivotal role and indeed they release distinct EVs carrying a complex range of signals directed to the surrounding cells. The EV-associated signals, by virtue of their specificity and diversity, are likely to contribute to finely orchestrate the regeneration process in a cooperative and concurring manner.
